# ClearF: a supervised feature scoring method to find biomarkers using class-wise embedding and reconstruction

**DOI:** 10.1186/s12920-019-0512-9

**Published:** 2019-07-11

**Authors:** Sehee Wang, Hyun-Hwan Jeong, Kyung-Ah Sohn

**Affiliations:** 1 Department of Computer Engineering, Ajou University, Suwon, 16499 South Korea; 2 Jan and Dan Duncan Neurological Research Institute, Texas Children’s Hospital, Houston, TX 77030 USA; 3 Department of Molecular and Human Genetics, Baylor College of Medicine, Houston, TX 77030 USA

**Keywords:** Feature selection, Feature scoring, Mutual information (MI), Breast cancer, Dimension reduction, Low-dimensional embedding, Reconstruction error, Principal component analysis (PCA)

## Abstract

**Background:**

Feature selection or scoring methods for the detection of biomarkers are essential in bioinformatics. Various feature selection methods have been developed for the detection of biomarkers, and several studies have employed information-theoretic approaches. However, most of these methods generally require a long processing time. In addition, information-theoretic methods discretize continuous features, which is a drawback that can lead to the loss of information.

**Results:**

In this paper, a novel supervised feature scoring method named ClearF is proposed. The proposed method is suitable for continuous-valued data, which is similar to the principle of feature selection using mutual information, with the added advantage of a reduced computation time. The proposed score calculation is motivated by the association between the reconstruction error and the information-theoretic measurement. Our method is based on class-wise low-dimensional embedding and the resulting reconstruction error. Given multi-class datasets such as a case-control study dataset, low-dimensional embedding is first applied to each class to obtain a compressed representation of the class, and also for the entire dataset. Reconstruction is then performed to calculate the error of each feature and the final score for each feature is defined in terms of the reconstruction errors. The correlation between the information theoretic measurement and the proposed method is demonstrated using a simulation. For performance validation, we compared the classification performance of the proposed method with those of various algorithms on benchmark datasets.

**Conclusions:**

The proposed method showed higher accuracy and lower execution time than the other established methods. Moreover, an experiment was conducted on the TCGA breast cancer dataset, and it was confirmed that the genes with the highest scores were highly associated with subtypes of breast cancer.

**Electronic supplementary material:**

The online version of this article (10.1186/s12920-019-0512-9) contains supplementary material, which is available to authorized users.

## Background

Feature selection or scoring techniques are essential for the solution of various problems in bioinformatics. Biomarkers are biological characteristics that can be used to predict the risks of diseases [1], and feature selection is a method used to detect them [2–4]. Various feature selection methods have been developed [5, 6] and successfully used to identify biomarkers. The feature selection method is also used to reduce large-scale data. The data used in bioinformatics generally contains a relatively small number of samples compared to the number of features. Thus, the ‘curse of dimensionality’ [7] easily occurs, in which the number of required samples exponentially increases as the number of features increases. To overcome this drawback, a feature selection method is often applied to the selection of important features. It is therefore important to develop feature selection algorithms for the detection of biomarkers.

With respect to labels, feature selection can be divided into two categories: 1) supervised feature selection methods that utilize class label information and 2) unsupervised methods that do not use class labels [8]. Supervised feature selection methods are used to find useful biomarkers for the prediction of disease. There are several categories of supervised feature selection methods [8]. For example, statistical based methods use statistical measures to score each feature, and similarity based approaches select important features that can preserve data similarity.

Information-theoretic methods perform a feature selection using mutual information, which is a measure of the entropy and conditional entropy dependence between a variable of data and a label: I (X; Y), The mutual information between two random variables X (a random variable of data, feature) and Y (a random variable of label) can be expressed as follows:

$$ I\left(X;Y\right)=H(X)-H\left(X|Y\right), $$

where H(X) is the entropy of the random variable X and H(X|Y) is the conditional entropy of the random variable of X given Y. Information theoretic approaches are typically used to detect biomarkers [9–15]. However, in most cases, the processing time is long. In addition, information theoretic methods discretize continuous variables, which is a drawback that can lead to loss of information [16].

While feature selection reduces the dimension by selecting a subset of the overall features, low-dimensional embedding is a method that creates new low-dimensional feature representations without preserving the original features. Low-dimensional embedding is often used to obtain a low-dimensional representation by its application to problems that are difficult to process at higher dimensions. It is also used for noise removal through reconstruction [17].

Principal component analysis (PCA) is a typical low-dimensional embedding method that uses an orthogonal linear transformation for a high-dimensional data to a low-dimensional representation. It offers a high execution speed, and it is frequently used in many fields. However, it does not reflect nonlinearity. The kernel principal component analysis (KernelPCA) [18] is an improvement of the original PCA using the kernel method. Another low-dimensional embedding method is the autoencoder, which is a specific type of neural network. Recently, deep neural networks have been established, and the deep autoencoder has been widely used.

Low-dimensional embedding is effective in reducing data to low dimensions; however, it constructs a set of new features, and it is difficult to extract substantial interpretations of these features in the transformed space. It is therefore difficult to use it directly for biomarker detection in conjunction with low-dimensional embedding. Furthermore, given that most of the methods are unsupervised, it is difficult to utilize class label information.

In this paper, we propose a method for assigning supervised feature scores by applying unsupervised class-wise low-dimensional embedding. The performance of the proposed method is in accordance with the principle of feature selection based on mutual information. Moreover, the method addresses the problems we described above.

## Methods

### Overview

**Fig. 1:**
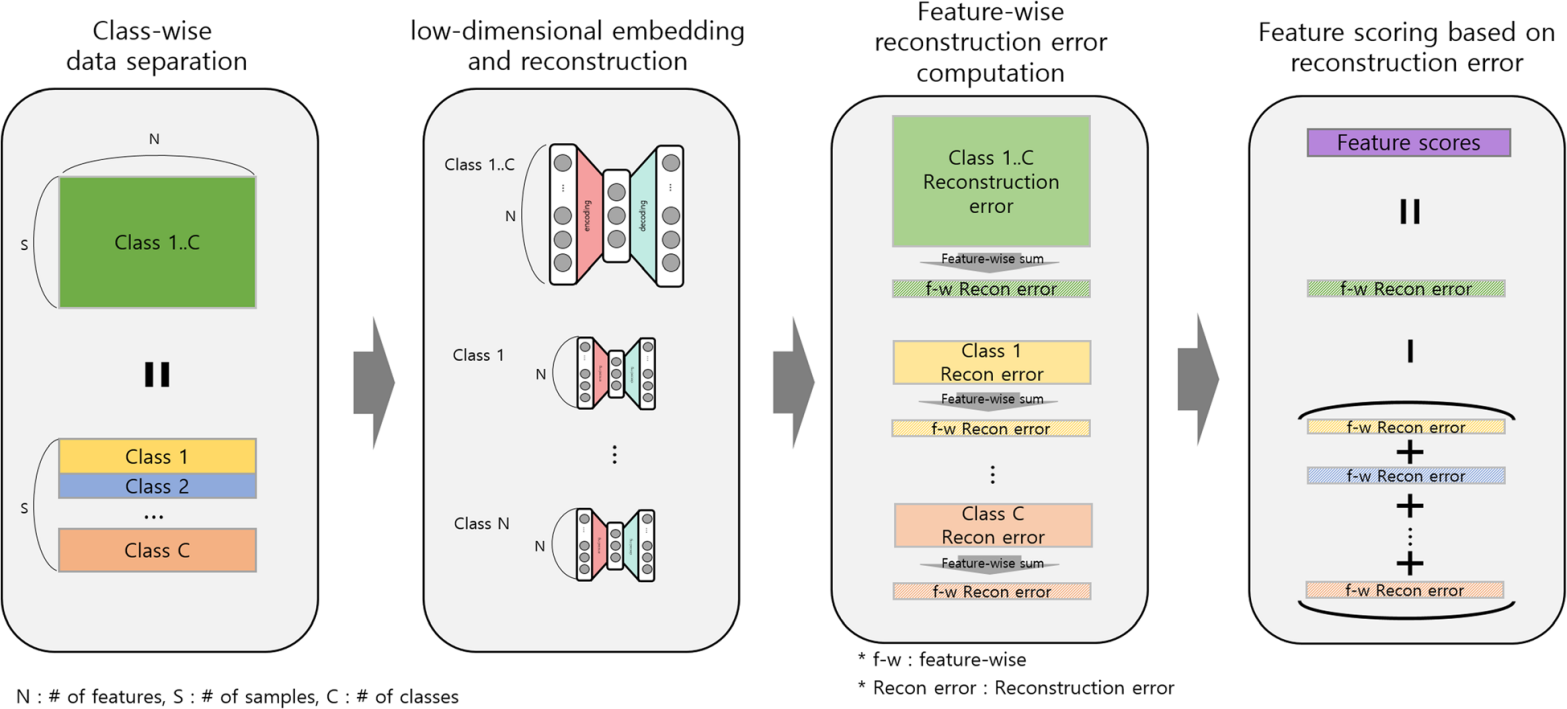
An overview of a supervised feature scoring method using class-wise embedding and reconstruction

**Fig. 2:**
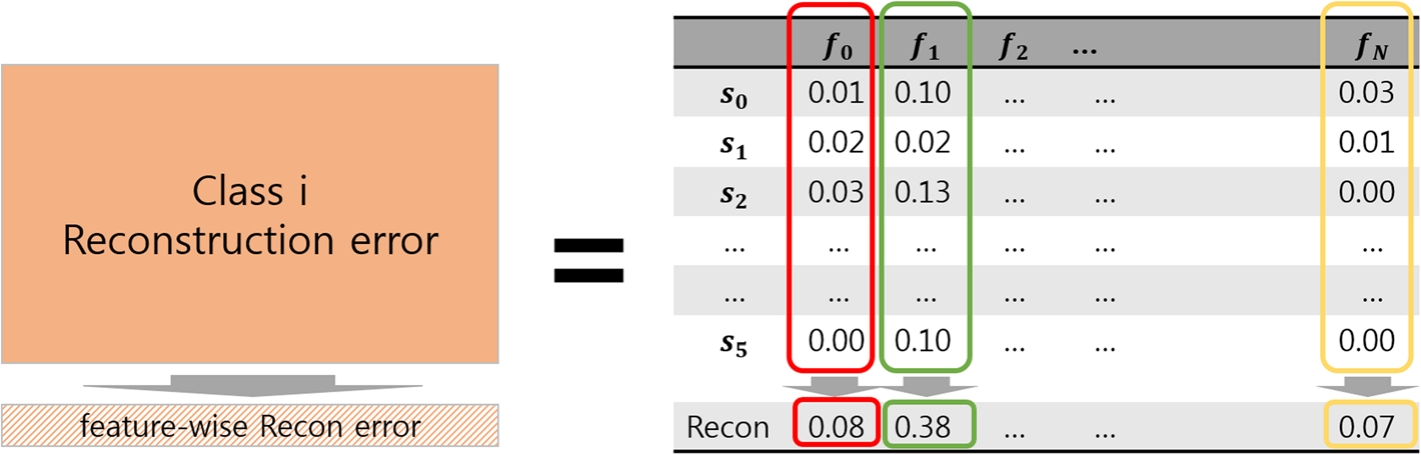
An illustration of feature-wise reconstruction error computation

The proposed method is termed ClearF. It determines the feature score by calculating the reconstruction error after class-wise low-dimensional embedding, and it uses the property of the reconstruction error that differs by class. Figure 1 presents the overall structure of ClearF. First, class-wise division is performed on the entire dataset. In other words, if the number of class labels is C, the entire dataset and data for each class are separated into C + 1 datasets. Thereafter, the low-dimensional embedding and reconstruction are separately performed for each divided dataset. Any low-dimensional embedding method such as PCA, KernelPCA, and autoencoder can be applied to ClearF. The reconstruction error for each dataset is obtained by calculating the difference between reconstructed data and original data. The feature-specific reconstruction errors in each dataset are calculated by the feature-wise sum of reconstruction errors for each feature (Fig. 2). Finally, the feature-wise reconstruction error of all the data and the error of data for each class are used to derive the final feature score.

### Conversion of mutual information to reconstruction error-based concept

Low-dimensional embedding reduces the size of high-dimensional data, and simplifies the data in the reduction process. Suppose we reduce a dataset to a very small dimension. If the characteristics of the data are complex, it is difficult to represent the characteristics of the original data. Thus, information may be lost in the low-dimensional embedding process. On the other hand, if the characteristics of the data are comparatively simple, they can be sufficiently reflected in the low dimension. Intuitively, this leads to the hypothesis that the data is complex if the reconstruction error is high; otherwise the data is simple.

**Fig. 3:**
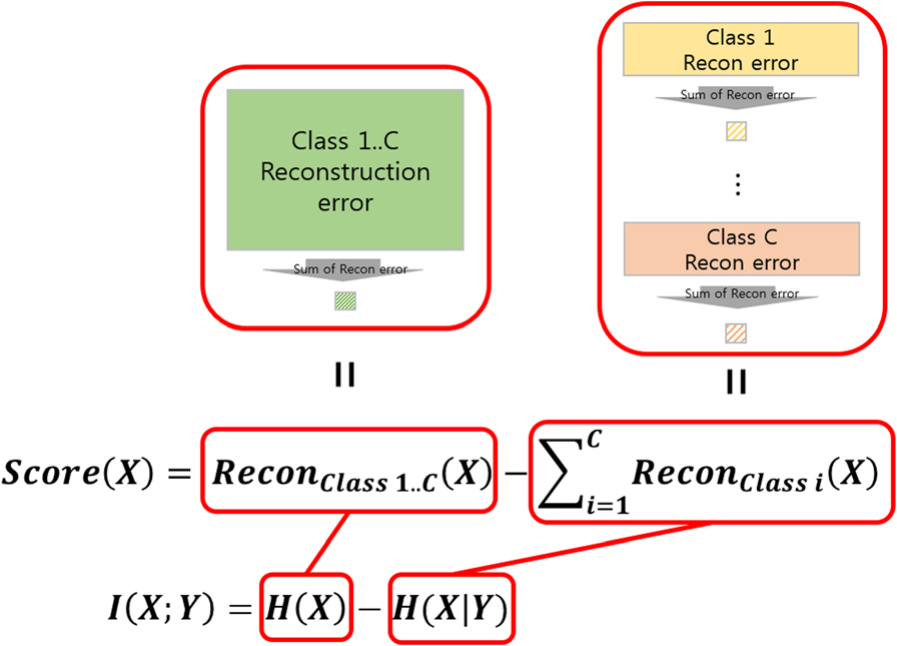
Conversion of a mutual information concept to a formula using the reconstruction error

In information theory, entropy refers to the uncertainty of the data, which increases in accordance with an increase in the complexity of the data. This is similar to the characteristics of the reconstruction error described above. We use this to express entropy as a reconstruction error in a low-dimensional embedding process. Figure 3 presents the relationship between terms in the score (X) and the entropy terms in the mutual information function. In particular, the entropy H(X) can be interpreted as the reconstruction error after the low-dimensional embedding of *X*. The conditional entropy H(X| Y) corresponds to the reconstruction error when each existing label information is expressed. In Fig. 3, *Recon*_*Class* 1. . *C*_(*X*) is a term of the reconstruction error after the low-dimensional embedding of data *X*; and ${\sum}_{i=0}^C{Recon}_{Class\ i}(X)$ is the sum of the terms applied to each class separately. That is, the first part is calculated without label information and corresponds to H(X). The latter part is a reconstruction error in the state given the label information, and it corresponds to the conditional entropy under the label information.

### Reconstruction error-based feature scoring

The reconstruction error of each feature in the feature-wise reconstruction error described in Fig. 2 is denoted as *Recon*_*Class i*_(*F*_*j*_). If it is associated with the score equation in Fig. 3, the following is obtained:

$$ {\displaystyle \begin{array}{c} Score(X)={Recon}_{Class\ 1..C}(X)-\sum \limits_1^C{Recon}_i(X)\kern0.5em \\ {}=\sum \limits_1^F{Recon}_{1..C}\left({F}_j\right)-\sum \limits_1^C\sum \limits_1^F{Recon}_i\left({F}_j\right)\\ {}=\sum \limits_1^F{Recon}_{1..C}\left({F}_j\right)-\sum \limits_1^F\sum \limits_1^C{Recon}_i\left({F}_j\right)\ \end{array}} $$

The total score of *X* is the sum of the scores calculated for each feature. The score calculated for each feature is the contribution of the feature to the total score; thus it can be called the score of the feature that can distinguish the label.

Our method can use any low-dimensional embedding method that is capable of reconstruction such as PCA, KernelPCA, and autoencoder. In the proposed method, the decreasing number of dimensions is a significant parameter. Since the purpose is to determine the reconstruction error difference of class-specific data with respect to that of the entire dataset, the same number of components or dimensions is used for the entire dataset and class-wise data.

## Results

### Correlations between the entropy and reconstruction error

To confirm the correspondence between the entropy and reconstruction error, a simulation was conducted.

The entropy of the multivariate Gaussian distribution can be calculated as follows, using the determinant of the covariance matrix [19]:

$$ H(X)=\frac{n}{2}+\frac{n}{2}\ln 2\pi +\ln \mid \varSigma \mid $$

where *n* is the number of features in X and Σ is the determinant of the covariance matrix. We used this to generate simulation data with a multivariate Gaussian distribution *N*(0, 1). Thereafter, the entropy was calculated to determine if there was a correlation with the reconstruction error.

**Fig. 4:**
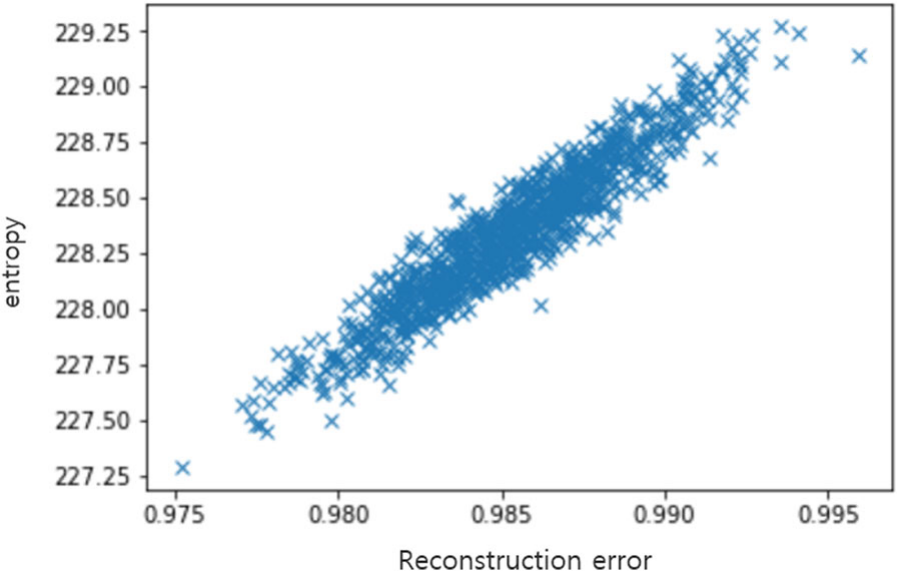
Simulation results of the relationship between the entropy and reconstruction error

The simulation data was generated to contain 100 features and 500 samples, and the entropy and reconstruction error of the generated data were calculated. Moreover, PCA was used for the low-dimensional embedding method, and the number of components in the PCA was set as one. We repeated this procedure 1000 times, and the results are presented in Fig. 4. The experimental results reveal that the entropy and the reconstruction error were highly correlated (*R*^2^ = 0.94).

### Simulation to verify the applicability of the proposed method

**Fig. 5:**
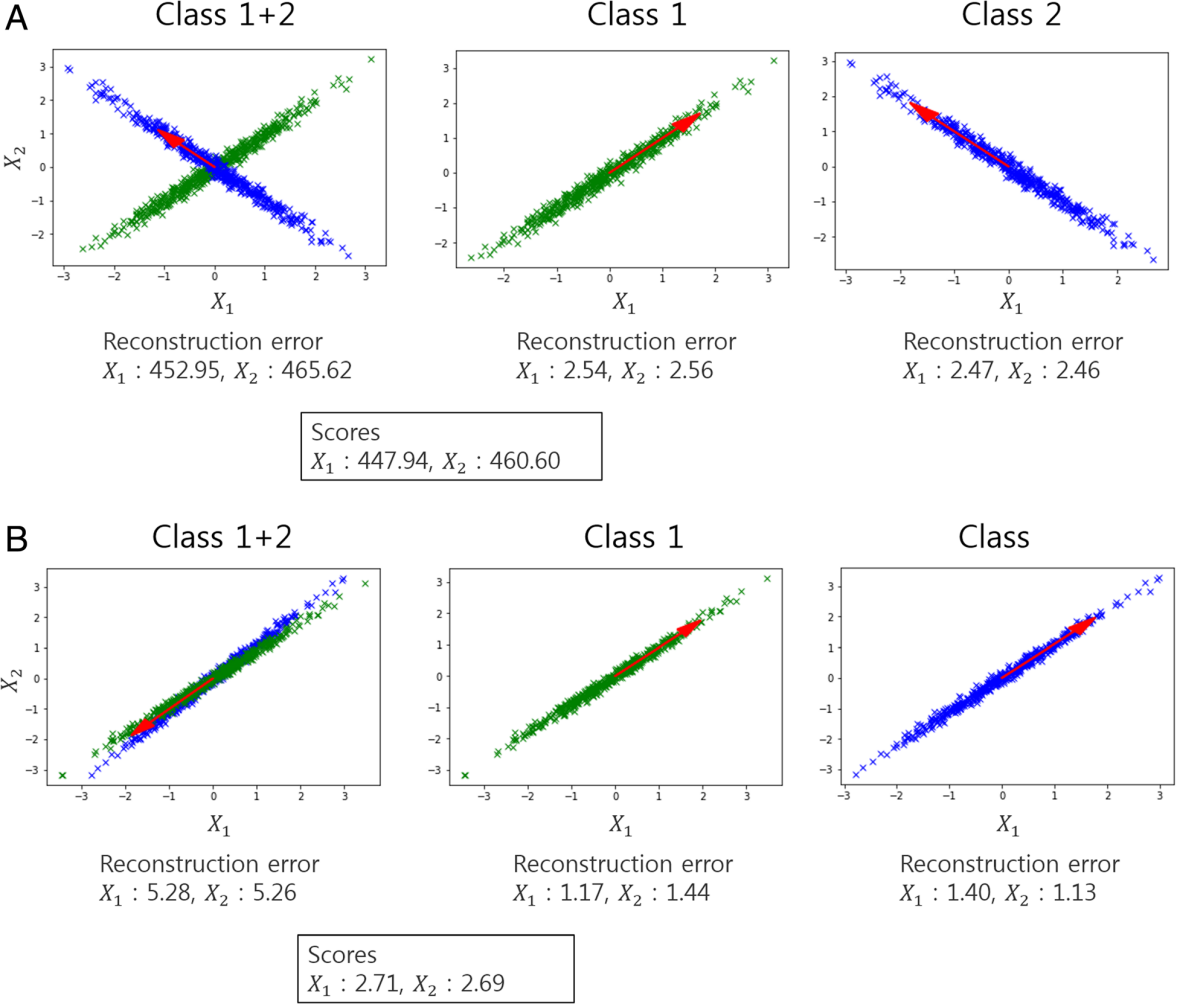
Simulation results confirming the applicability of the scoring method for feature selection. The red arrows indicate the direction and size of the components. **a** shows the result on the dataset with two features that differ widely between classes, and **b** shows the result when there is little difference between classes

We performed an experiment to confirm that our method works properly with a simulated dataset. The experiment was conducted to evaluate each score in the following two cases: 1) when there is a large difference between the data for each class, and 2) when there is no difference between the data for each class. As shown in Fig. 5, we created two different datasets with two features and 500 samples. In particular, A was a dataset that contained two features that represented different trends by class, and B was a dataset that contained two features with fewer differences between classes. As in the previous experiments, PCA was used for the low-dimensional embedding, and the number of components in the PCA was set as one.

As shown in Fig. 5a, if combination of two features (*X*_1_ and *X*_2_ in the figure) can easily differentiate data into the two classes, the reconstruction error for each class is very low. However, the reconstruction error of the entire dataset was very large; thus, the scores were 447.94 and 460.60, respectively, for each feature. On the other hand, in the case of Fig. 5b, the reconstruction error of the entire dataset was small; which resulted in low scores of 2.71 and 2.69, respectively. These results reveal that our scoring method produces high scores for the features that differ by class, and low when there is no difference by class.

### Performance validation for benchmark datasets

To compare the performances of the proposed method and other established methods, five biological datasets were used from the benchmark datasets in [8]. Table 1 shows the details of the datasets. Several feature selection algorithms were used to conduct the benchmark tests. For performance comparison, we chose commonly used feature selection algorithms from each category described above. The selected algorithms were t-score [20] (binary class only), CMIM [21], mRMR [22] (discrete data only), Fisher score [23], Trace ratio [24], and multi-SURF [25]. For MultiSURF, we used the code provided by ReBATE [25], and the remaining algorithms were compared using code from scikit-features [8].

**Table 1:** Detailed information of benchmark datasets

Data set	Data type	Number of classes	Number of features	Number of samples	Data information
Leukemia	Discrete	2	7070	72	SNP
ProstateGE	Continuous	2	5966	102	Gene expression
TOX171	Continuous	4	5748	171	Gene expression
Lung	Continuous	5	3312	203	Gene expression
LungDiscrete	Discrete	7	325	73	SNP

In order to confirm that the proposed method extracts more effective features for class label classification, the selected features from each method were used for classification, and the resulting accuracies were compared. We conducted a 10-fold cross validation in which the entire dataset was divided into 10 folds, one for test data and the other for training data. The feature selection algorithm was applied only to the training data to extract important features. The classification algorithm of support vector machine (SVM) with radial basis function (RBF) kernel was applied using only the selected features, and the average accuracy of the 10-fold cross validation was measured. The number of features to be selected in the above procedure was increased by five, and the process was the same as that used in the algorithm comparison in the previous study [8].

In our experimental setting, the low-dimensional embedding methods of PCA and KernelPCA were used. The kernels used for KernelPCA were the RBF kernel and the polynomial kernel with degree of three. Given that the component size is an important hyperparameter in the proposed method, we used a greedy search algorithm to find the optimal component size. The component size was selected using only training data in each of 10 measurements of 10-fold cross validation. First, the training data is further divided into three equal-sized subsets, two of which are used for model training and one of which is set as validation data. Let *C*_*min*_ be the number of samples of the smallest class in the dataset. We applied our method to training data for five different component sizes (1, *C*_*min*_/4, *C*_*min*_/2, 3 ∙ *C*_*min*_/4 and *C*_*min*_). We selected the component size with the highest accuracy by applying SVM classifier. The mean squared error was calculated to measure the reconstruction error.

**Fig. 6:**
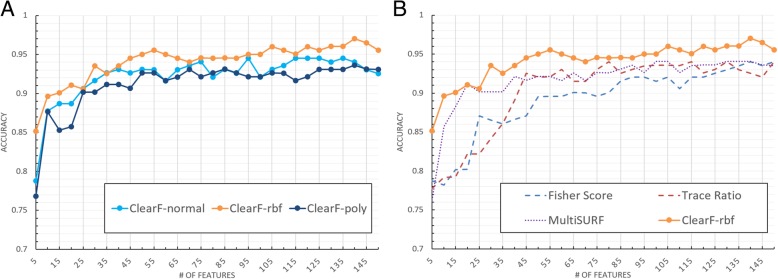
Cross-validation accuracy for the Lung dataset with respect to the number of selected features. **a** presents the results of the PCA (ClearF-normal), KernelPCA with RBF kernel (ClearF-rbf) and KernelPCA with polynomial kernel (ClearF-poly) used in the proposed method; and **b** compares the results of the other algorithms with our method using the best result kernel

Figure 6 shows the result for the Lung dataset. We have performed the benchmarking using the previously mentioned algorithms, with the exception of T-score (only applicable to the binary class), mRMR, and CMIM (only applicable to discrete data), because the Lung dataset is a continuous and multi-class dataset. The proposed method demonstrated a relatively superior performance to those of the other algorithms. Especially, the RBF kernel showed a better performance than other methods regardless of the number of selected features.

**Fig. 7:**
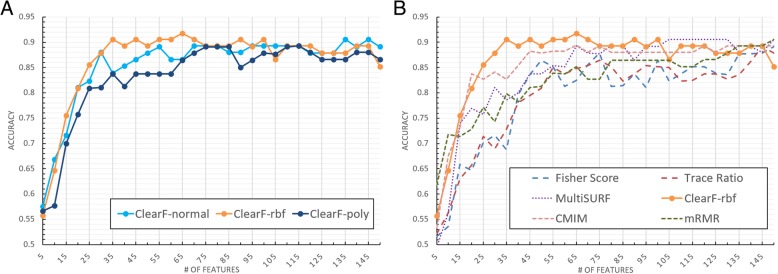
Cross-validation accuracy for the LungDiscrete dataset with respect to the number of selected features. **a** presents the results of the PCA (ClearF-normal), KernelPCA with RBF kernel (ClearF-rbf) and KernelPCA with polynomial kernel (ClearF-poly) used in the proposed method; and **b** compares the results of the other algorithms with our method using the best result kernel

The results for the LungDiscrete dataset are shown in Fig. 7. We excluded T-score because this dataset is a multi-class dataset. When the number of features was very small (> 20), mRMR and CMIM demonstrated comparable performances. However, when the number of features was larger than 30, ClearF-RBF archived a higher accuracy than other methods.

**Fig. 8:**
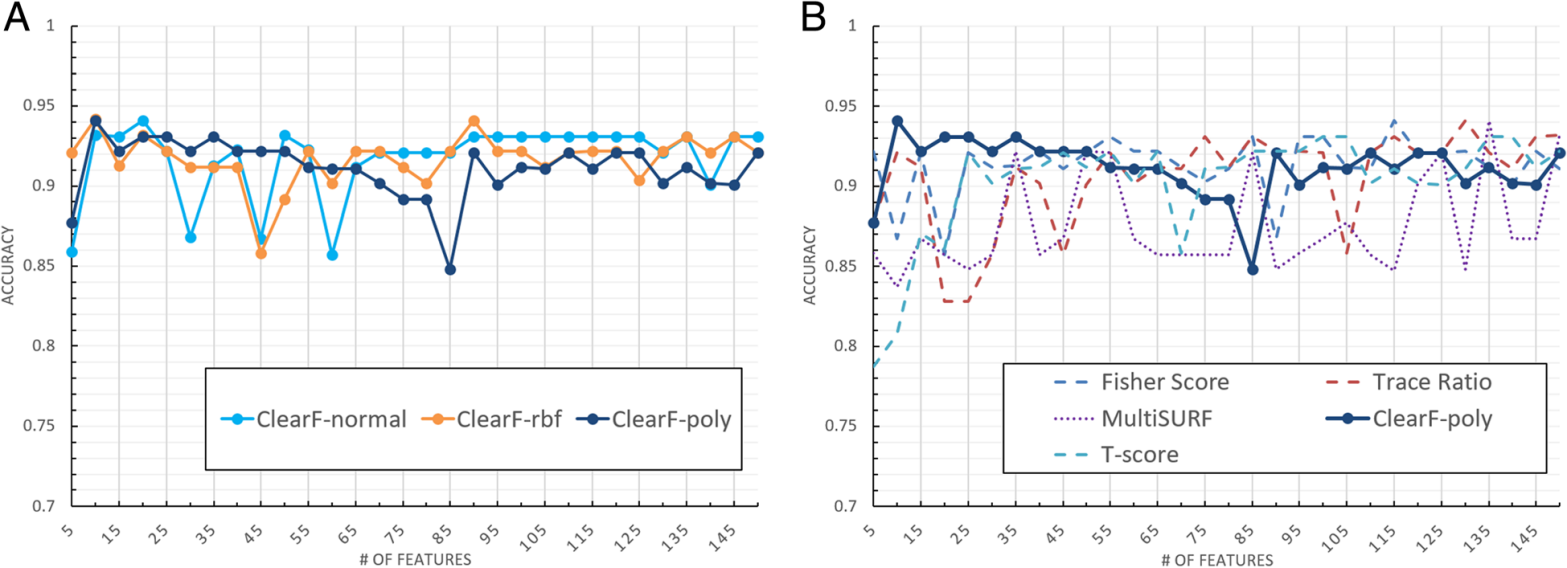
Cross-validation accuracy for the ProstateGE dataset with respect to the number of features. **a** presents the results of the PCA (ClearF-normal), KernelPCA with RBF kernel (ClearF-rbf) and KernelPCA with polynomial kernel (ClearF-poly) used in the proposed method; and **b** compares the results of the other algorithms with our method using the best result kernel

Figure 8 presents the results for the ProstateGE dataset. In the cases of using 10–30 features, the proposed method showed better performance than the other algorithms. The results of using more than 30 features were almost identical with the accuracy close to 0.9.

The results for the Leukemia dataset are presented in Additional file 1: Figure S1. Most of all the methods demonstrated accuracies higher than 0.95, and no significant difference between the performances of the methods were observed. In Additional file 1: Figure S2 presents the result of the Tox171 dataset. Although the results of the MultiSURF were relatively more accurate than those of the other methods, the results of our method using PCA were comparable.

For the purposes of feature selection, it is important that even a small number of selected features yield good results. The average accuracies for each method, from 5 to 50 features, are presented in Table 2. The bold italic numbers indicate the best results for each dataset, and the bold non-italic numbers indicate the second-best result. The results show that the proposed method mostly showed a good performance. All the 10-fold cross validation accuracies and their standard deviations are shown in Additional file 1: Tables S1, S2, S3, S4, S5 and S6.

**Table 2:** Average accuracy of using 5 to 50 features per method and dataset

	Fisher score	Trace ratio	Multi SURF	ClearF normal	ClearF rbf	ClearF poly	CMIM	mRMR	t-score
Leukemia	0.945	0.945	0.945	***0.973***	0.959	0.945	***0.973***	0.959	0.959
ProstateGE	0.912	0.857	0.857	0.868	0.912	***0.922***	–	–	0.902
TOX171	0.672	0.713	***0.819***	**0.807**	0.666	0.683	–	–	–
Lung	0.865	0.841	0.901	**0.916**	***0.935***	0.902	–	–	–
LungDiscrete	0.716	0.689	0.811	***0.880***	**0.879**	0.811	0.841	0.743	–

The bold italic numbers indicate the best results for each dataset, and the bold non-italic numbers indicate the second-best result

### Computational cost validation for benchmark datasets

A comparison of the computational costs was performed to show that the proposed method has advantages of a reduced execution time. Among the benchmark datasets, LungDiscrete and ProstateGE were used, and the CPU time was measured by running each method 10 times for each data. In the case of MultiSURF, since it provides multi core implementation in ReBATE [25] code, separate experiments were conducted using a single core and six cores.

**Fig. 9:**
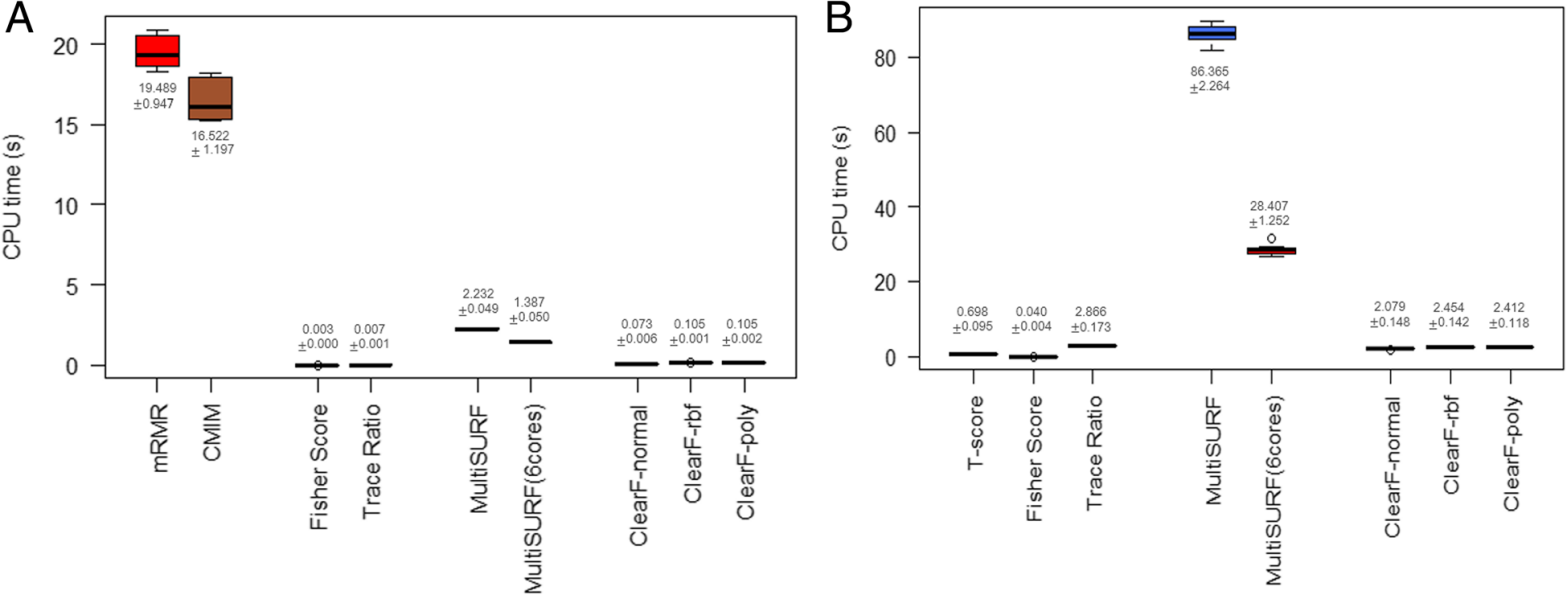
Comparison of execution times for the LungDiscrete (**a**) and ProstatGE (**b**) dataset

Figure 9a presents the results for the LungDiscrete dataset. It can be seen that the execution times of CMIM and mRMR, which are information-theoretic based methods, were much higher than those of the other methods. The results for the ProstateGE dataset are shown in Fig. 9b. The execution times of the other methods were lower than that of MultiSURF. The running time of our method is significantly shorter than those of the other feature selection methods except for the simple T-score or Fisher Score.

### Performance validation for the TCGA breast cancer dataset

We performed an experiment using the TCGA (The Cancer Genome Atlas) gene expression data of patients with breast cancer. The genes with missing data were removed, and tested with 13,615 genes and 389 patients’ data. Among the samples, 15 patients had HER2 positive, 280 had Luminal A, 37 had Luminal B, and 57 had basal-like subtypes.

**Fig. 10:**
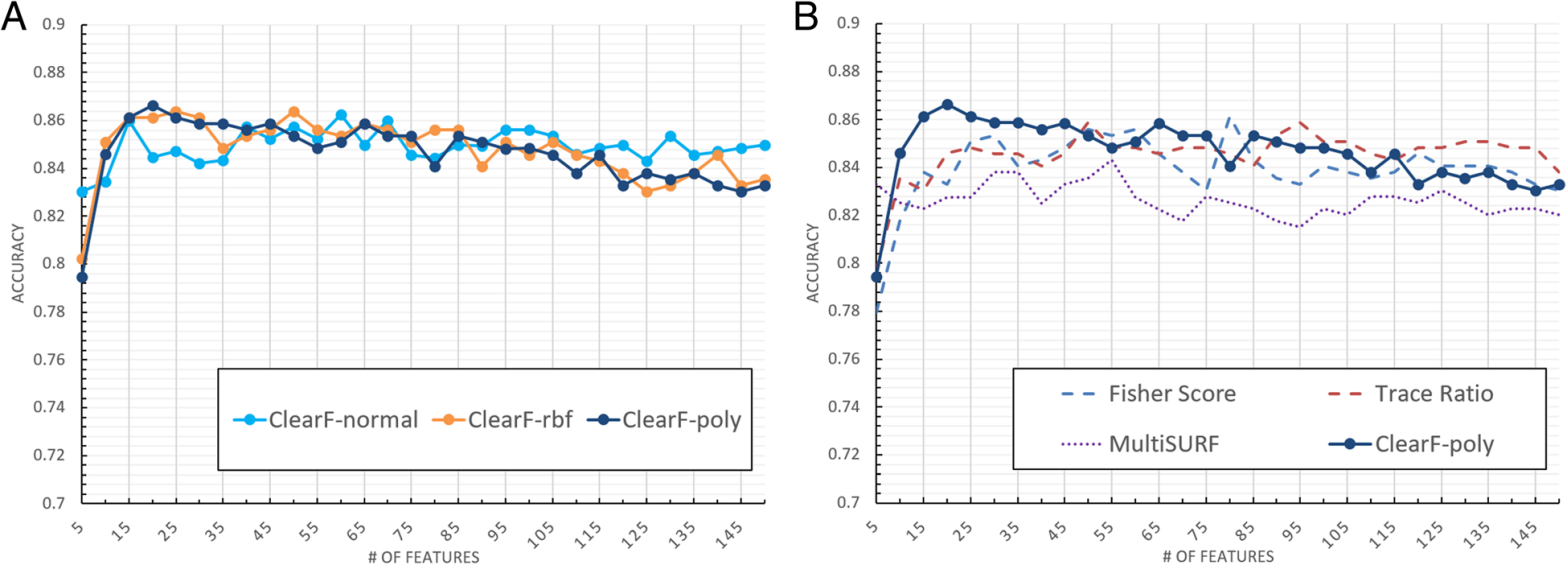
Cross-validation accuracy for the TCGA dataset with respect to the number of features. **a** presents the results of the PCA (ClearF-normal), KernelPCA with RBF kernel (ClearF-rbf) and KernelPCA with polynomial kernel (ClearF-poly) used in the proposed method; and **b** compares the results of the other algorithms with our method using the best result kernel

Accuracy tests were performed on the TCGA breast cancer dataset in the same manner as the experiment in the benchmark dataset. The results are shown in Fig. 10, and they reveal that the proposed method yielded the best results for most of the sections.

Our method based on the KernelPCA (with polynomial kernel), which showed a good performance in the performance evaluation, was applied to all the data. The 30 genes with the highest score are presented in Table 3.

**Table 3:** The top 30 genes with the highest scores obtained from the TCGA dataset

Rank	Gene symbol	Entrez Gene Id	Gene Description	Score
1	ERBB2	2064	v-erb-b2 erythroblastic leukemia viral oncogene homolog 2, neuro/glioblastoma derived oncogene homolog (avian)	0.416
2	STARD3	10,948	StAR-related lipid transfer (START) domain containing 3	0.339
3	PGAP3	93,210	post-GPI attachment to proteins 3	0.295
4	FOXC1	2296	forkhead box C1	0.276
5	CDKN2A	1029	cyclin-dependent kinase inhibitor 2A (melanoma, p16, inhibits CDK4)	0.270
6	ORMDL3	94,103	ORM1-like 3 (*S. cerevisiae*)	0.259
7	GSDMB	55,876	gasdermin B	0.245
8	B3GNT5	84,002	UDP-GlcNAc:betaGal beta-1,3-N-acetylglucosaminyltransferase 5	0.236
9	PSMD3	5709	proteasome (prosome, macropain) 26S subunit, non-ATPase, 3	0.235
10	HAPLN3	145,864	hyaluronan and proteoglycan link protein 3	0.231
11	CDCA7	83,879	cell division cycle associated 7	0.222
12	PSAT1	29,968	phosphoserine aminotransferase 1	0.216
13	C17orf37	84,299	migration and invasion enhancer 1	0.215
14	GABRP	2568	gamma-aminobutyric acid (GABA) A receptor, pi	0.215
15	TMSB15B	286,527	thymosin beta 15B	0.214
16	MED1	5469	mediator complex subunit 1	0.208
17	CDCA2	157,313	cell division cycle associated 2	0.207
18	FAM171A1	221,061	family with sequence similarity 171, member A1	0.203
19	CCNE1	898	cyclin E1	0.197
20	CDK12	51,755	cyclin-dependent kinase 12	0.194
21	DSC2	1824	desmocollin 2	0.192
22	STAC	6769	SH3 and cysteine rich domain	0.189
23	PADI2	11,240	peptidyl arginine deiminase, type II	0.189
24	RCOR2	283,248	REST corepressor 2	0.179
25	IGF2BP2	10,644	insulin-like growth factor 2 mRNA binding protein 2	0.176
26	CDH3	1001	cadherin 3, type 1, P-cadherin (placental)	0.175
27	ZNF695	57,116	zinc finger protein 695	0.175
28	CLCN4	1183	chloride channel 4	0.172
29	MEX3A	92,312	mex-3 homolog A (*C. elegans*)	0.171
30	CBS	875	cystathionine-beta-synthase	0.171

## Discussion

Given the purpose of feature selection to identify important biomarkers, it is essential for a feature selection method to show good performance in selecting a small number of meaningful features. For the TCGA dataset as well as the benchmark datasets, the proposed method performed better than the other algorithms, especially in the results of selecting a small number of features (10 ~ 50), which demonstrates the utility of our method for biomarker identification.

Among the highest-scoring genes detected using the proposed method, it is suspected that STARD3, PGAP3, ORMDL3, PSMD3 and HAPLN3 are the biomarkers of the HER2 + subtype [26–31], thus indicating that FOXC1 can identify basal-like subtypes in hereditary breast cancer cohorts [32]. In addition, the methylation status of CDKN2A exon2 has markedly higher methylation levels in luminal A and luminal B subtypes [33]. Moreover, gene expression may be a potential biomarker, as it is associated with methylation levels. B3GNT5 expression can also be a measure that distinguishes the subtypes luminal A, B from basal-like as it is previously reported that the expression in basal-like samples is high, while the expression in luminal A and B is significantly lower [34].

Furthermore, GABRP, STAC, RCOR2, and IGFBP2 are the genes scored high by our method, but not by the other algorithms used in the performance comparison. The expression of GABA (A) receptor pi (GABRP) plays a role in initiation and progression of basal-like tumors, and has therapeutic potential in basal-like breast cancer [35]. In addition, the overexpression of IGFBP2 may be a feature of basal-like breast cancer that correlates with a low survival rate [36]. Moreover, RCOR2 replaces the need for Sox2 expression in somatic cell reprogramming [37], whereas Sox2 has a positive expression in the basal-like subtype [38], although it has not been directly related to the breast cancer subtype. The similarity between somatic cell reprogramming and tumorigenesis [39] may suggest that Rcor2 is a potential biomarker.

**Fig. 11:**
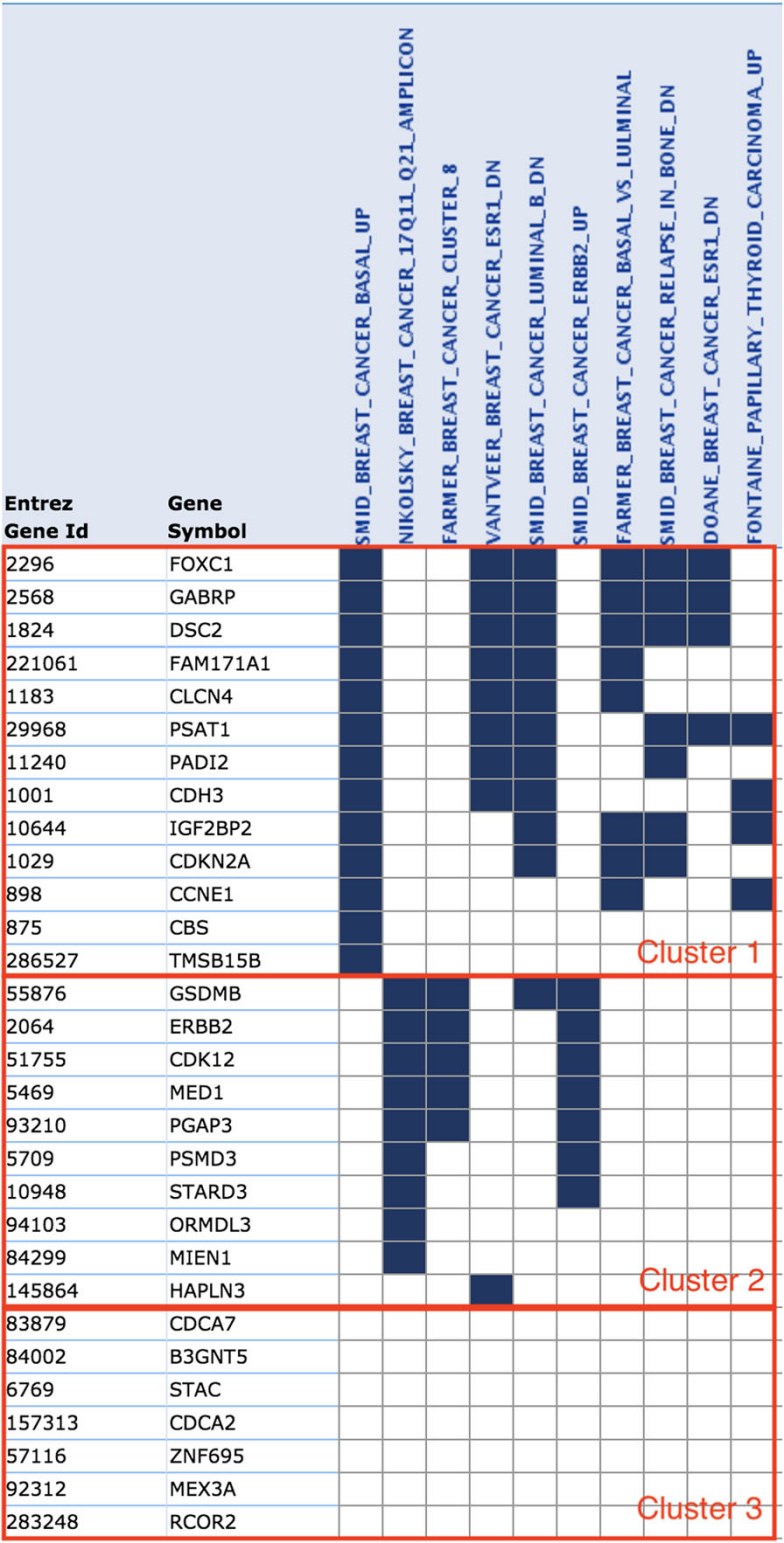
Cluster information based on overlap of MsigDB and Selected Genes

We have also checked which of the top 30 genes belong to the C2 collection (curated gene sets) of MSigDB [40, 41]. The results are described in Table 4 and Fig. 11. Using the information in Table 4, genes can be classified into three clusters as shown in Fig. 11. As we have seen individually above, we find that Cluster 1 belongs to the up-regulated gene set in the basal-like subtype, and Cluster 2 is included in the Her2 + (triple negative) gene set.

**Table 4:** Significant gene sets of overlap between MSigDB and Selected Genes

Gene Set Name (# Genes)	Description	# Genes in Overlap	*p*-value	FDR q-value
SMID_BREAST_CANCER_BASAL_UP (648)	Genes up-regulated in basal subtype of breast cancer samples.	13	7.43 e-17	7.85 e-13
NIKOLSKY_BREAST_CANCER_17Q11_Q21_AMPLIPLICON (133)	Genes within amplicon 17q11-q21 identified in a copy number alterations study of 191 breast tumor samples.	9	1.47 e-16	7.85 e-13
FARMER_BREAST_CANCER_CLUSTER_8 (7)	Cluster 8: selected ERBB2 (GeneID = 2064) amplicon genes clustered together across breast cancer samples.	5	1.75 e-15	6.23 e-12
VANTVEER_BREAST_CANCER_ESR1_DN (240)	Down-regulated genes from the optimal set of 550 markers discriminating breast cancer samples by ESR1 (GeneID = 2099) expression: ER(+) vs ER(−) tumors.	9	3.23 e-14	8.16 e-11
SMID_BREAST_CANCER_LUMINAL_B_DN (564)	Genes down-regulated in the luminal B subtype of breast cancer.	11	3.82 e-14	8.16 e-11
SMID_BREAST_CANCER_ERBB2_UP (147)	Genes up-regulated in the erbb2 subype of breast cancer samples, characterized by higher expression of ERBB2 (GeneID = 2064).	7	5.68 e-12	1.01 e-8
FARMER_BREAST_CANCER_BASAL_VS_LULMINAL (330)	Genes which best discriminated between two groups of breast cancer according to the status of ESR1 and AR (GeneID = 2099;367): basal (ESR1- AR-) and luminal (ESR1+ AR+).	8	3.31 e-11	5.05 e-8
SMID_BREAST_CANCER_RELAPSE_IN_BONE_DN (315)	Genes down-regulated in bone relapse of breast cancer.	7	1.18 e-9	1.58 e-6
DOANE_BREAST_CANCER_ESR1_DN (48)	Genes down-regulated in breast cancer samples positive for ESR1 (GeneID = 2099) compared to the ESR1 negative tumors.	4	2.81 e-8	3.34 e-5
FONTAINE_PAPILLARY_THYROID_CARCINOMA_UA_UP (66)	Genes up-regulated in papillary thyroid carcinoma (PTC) compared to other thyroid tumors.	4	1.03 e-7	1.1 e-4

Although the genes in Cluster 3 are not part of a significant gene set, CDCA2 and CDCA7 are genes involved in the cell division cycle-associated protein. Expression of several genes involved in cell division cycle-associated protein has been reported to cause shorter relapse free survival in patients with breast cancer [42]. Therefore, CDCA2 and CDCA7 may also be potential biomarkers. In addition, as previously described, Rcor2 is likely to be a potential biomarker, so other genes in Cluster 3 may also be potential biomarkers.

## Conclusion

In this study, we developed a supervised feature selection algorithm that extracts useful features for the prediction of diseases or subtypes in biological data. By conducting simulation, we showed the applicability of the proposed method for feature selection. The experimental results revealed that our method has advantages both in terms of classification accuracy and execution speed, and is therefore useful in detecting biomarkers. This was also demonstrated by the extraction of meaningful genes and gene sets when applied to the TCGA dataset.

Additionally, we tried to use an auto-encoder for low-dimensional embedding, but the results were not stable. It is possible that the size of the dataset was not sufficiently big to show reasonable performance. In future work, the performance of an auto-encoder or other embedding methods should be evaluated using an appropriately sized datasets. In addition, we plan to conduct further studies on the selection of component numbers and their effects on the performance.

## Additional file

Additional file 1: **Figure S1.** Cross-validation accuracy for the Leukemia dataset with respect to the number of features. A presents the results of the PCA (ClearF-normal), KernelPCA with RBF kernel (ClearF-rbf) and KernelPCA with polynomial kernel (ClearF-poly) used in the proposed method; and B compares the results of the other algorithms with our method using the best result kernel. **Figure S2.** Cross-validation accuracy for the TOX171 dataset with respect to the number of features. A presents the results of the PCA (ClearF-normal), KernelPCA with RBF kernel (ClearF-rbf) and KernelPCA with polynomial kernel (ClearF-poly) used in the proposed method; and B compares the results of the other algorithms with our method using the best result kernel. **Table S1.** Detailed results of performance validation for Lung dataset. **Table S2.** Detailed results of performance validation for LungDiscrete dataset. **Table S3.** Detailed results of performance validation for ProstateGE dataset. **Table S4.** Detailed results of performance validation for Leukemia dataset. **Table S5.** Detailed results of performance validation for TOX171 dataset. **Table S6.**Detailed results of performance validation for TCGA dataset. (PDF 437 kb)

## Abbreviations

MI: Mutual information
PCA: Principal component analysis
TCGA: The cancer genome atlas
