# Supplementary material for: ClearF: a supervised feature scoring method to find biomarkers using class-wise embedding and reconstruction
Source: BMC Med Genomics. 2019 Jul 11;12(Suppl 5):95. doi: 10.1186/s12920-019-0512-9 (PMC6624178; doi:10.1186/s12920-019-0512-9)
Supplement: Supplementary file 1 — Figure S1. Cross-validation accuracy for the Leukemia dataset with respect to the number of features. A presents the results of the PCA (ClearF-normal), KernelPCA with RBF kernel (ClearF-rbf) and KernelPCA with polynomial kernel (ClearF-poly) used in the proposed method; and B compares the results of the other algorithms with our method using the best result kernel. Figure S2. Cross-validation accuracy for the TOX171 dataset with respect to the number of features. A presents the results of the PCA (ClearF-normal), KernelPCA with RBF kernel (ClearF-rbf) and KernelPCA with polynomial kernel (ClearF-poly) used in the proposed method; and B compares the results of the other algorithms with our method using the best result kernel. Table S1. Detailed results of performance validation for Lung dataset. Table S2. Detailed results of performance validation for LungDiscrete dataset. Table S3. Detailed results of performance validation for ProstateGE dataset. Table S4. Detailed results of performance validation for Leukemia dataset. Table S5. Detailed results of performance validation for TOX171 dataset. Table S6.Detailed results of performance validation for TCGA dataset. (PDF 437 kb) [file 12920_2019_512_MOESM1_ESM.pdf]

Supplementary materials

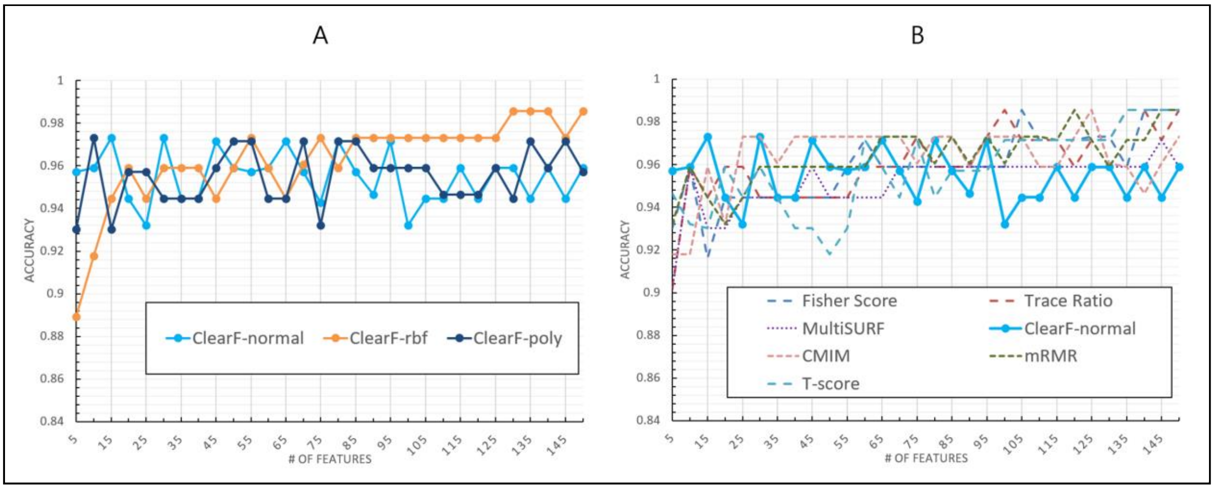

Figure S1 - Cross-validation accuracy for the Leukemia dataset with respect to the number of features. A presents the results of the PCA (ClearF-normal), KernelPCA with RBF kernel (ClearF-rbf) and KernelPCA with polynomial kernel (ClearF-poly) used in the proposed method; and B compares the results of the other algorithms with our method using the best result kernel.

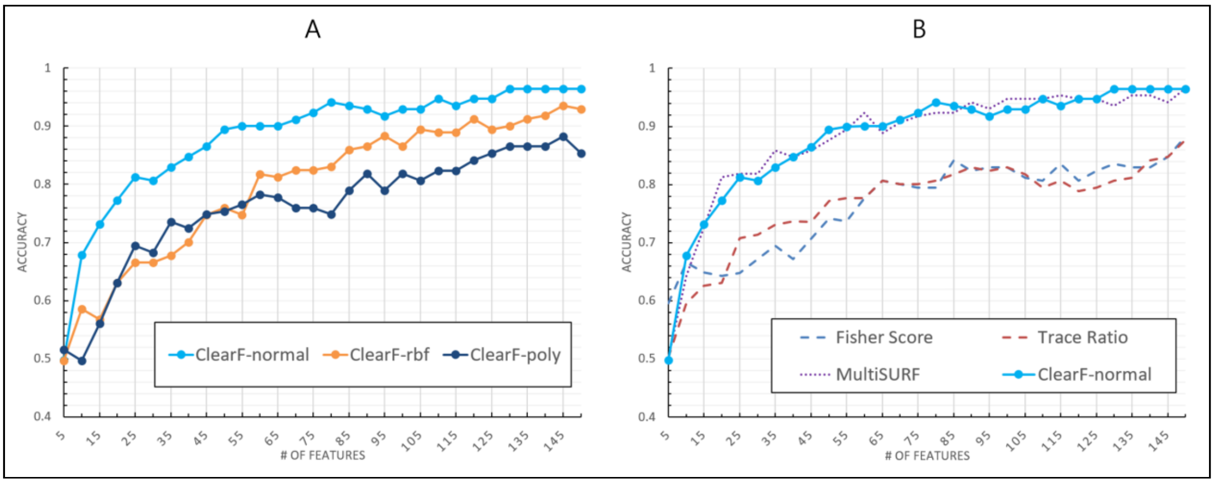

Figure S2 - Cross-validation accuracy for the TOX171 dataset with respect to the number of features. A presents the results of the PCA (ClearF-normal), KernelPCA with RBF kernel (ClearF-rbf) and KernelPCA with polynomial kernel (ClearF-poly) used in the proposed method; and B compares the results of the other algorithms with our method using the best result kernel.

Table S1 - Detailed results of performance validation for Lung dataset

# of features	Fisher score	Trace ratio	Multi SURF	ClearF normal	ClearF rbf	ClearF poly
5	0.79±0.07	0.78±0.06	0.76±0.12	0.79±0.1	0.85±0.07	0.77±0.06
10	0.78±0.08	0.79±0.08	0.86±0.06	0.88±0.11	0.9±0.08	0.88±0.09
15	0.8±0.09	0.79±0.07	0.88±0.08	0.89±0.08	0.9±0.09	0.85±0.08
20	0.8±0.1	0.82±0.09	0.91±0.06	0.89±0.09	0.91±0.11	0.86±0.08
25	0.87±0.12	0.82±0.08	0.9±0.06	0.91±0.07	0.91±0.1	0.9±0.08
30	0.87±0.1	0.84±0.08	0.9±0.05	0.92±0.08	0.94±0.08	0.9±0.07
35	0.86±0.09	0.86±0.1	0.9±0.07	0.93±0.07	0.93±0.07	0.91±0.06
40	0.87±0.1	0.89±0.1	0.92±0.06	0.93±0.04	0.94±0.07	0.91±0.08
45	0.87±0.09	0.93±0.07	0.92±0.06	0.93±0.07	0.95±0.07	0.91±0.08
50	0.9±0.07	0.92±0.08	0.92±0.06	0.93±0.07	0.95±0.07	0.93±0.09
55	0.9±0.07	0.92±0.06	0.92±0.07	0.93±0.06	0.96±0.06	0.93±0.08
60	0.9±0.08	0.93±0.06	0.92±0.07	0.92±0.06	0.95±0.07	0.92±0.09
65	0.9±0.07	0.92±0.07	0.93±0.07	0.93±0.06	0.95±0.07	0.92±0.08
70	0.9±0.09	0.92±0.08	0.92±0.07	0.94±0.06	0.94±0.07	0.93±0.08
75	0.9±0.09	0.93±0.08	0.93±0.07	0.94±0.07	0.95±0.06	0.92±0.07
80	0.9±0.09	0.94±0.07	0.93±0.08	0.92±0.06	0.95±0.06	0.93±0.09
85	0.92±0.09	0.93±0.08	0.93±0.08	0.93±0.06	0.95±0.06	0.93±0.08
90	0.92±0.1	0.93±0.07	0.94±0.07	0.93±0.08	0.95±0.07	0.93±0.08
95	0.92±0.1	0.94±0.07	0.93±0.07	0.95±0.07	0.95±0.07	0.92±0.07
100	0.92±0.12	0.94±0.07	0.94±0.06	0.92±0.07	0.95±0.06	0.92±0.08
105	0.92±0.09	0.94±0.08	0.94±0.06	0.93±0.07	0.96±0.07	0.93±0.07
110	0.91±0.1	0.94±0.08	0.93±0.06	0.94±0.07	0.96±0.06	0.93±0.08
115	0.92±0.09	0.94±0.07	0.94±0.06	0.95±0.07	0.95±0.06	0.92±0.07
120	0.92±0.09	0.93±0.07	0.94±0.06	0.94±0.08	0.96±0.07	0.92±0.07
125	0.93±0.08	0.93±0.07	0.94±0.06	0.94±0.08	0.96±0.07	0.93±0.08
130	0.93±0.06	0.94±0.09	0.94±0.06	0.94±0.08	0.96±0.07	0.93±0.08
135	0.94±0.06	0.93±0.08	0.94±0.06	0.94±0.08	0.96±0.06	0.93±0.08
140	0.94±0.08	0.93±0.07	0.94±0.06	0.94±0.08	0.97±0.07	0.94±0.08
145	0.94±0.08	0.92±0.08	0.94±0.06	0.93±0.08	0.96±0.06	0.93±0.08
150	0.94±0.08	0.94±0.08	0.94±0.06	0.93±0.08	0.96±0.06	0.93±0.07

Table S2 - Detailed results of performance validation for LungDiscrete dataset

# of features	mRMR	CMIM	Fisher score	Trace ratio	Multi SURF	ClearF normal	ClearF rbf	ClearF poly
5	0.62±0.2	0.53±0.23	0.52±0.19	0.52±0.15	0.49±0.13	0.57±0.21	0.56±0.21	0.57±0.18
10	0.72±0.15	0.68±0.22	0.54±0.09	0.56±0.11	0.56±0.16	0.67±0.16	0.65±0.13	0.58±0.18
15	0.71±0.18	0.73±0.13	0.66±0.14	0.63±0.14	0.74±0.13	0.72±0.19	0.76±0.15	0.7±0.18
20	0.73±0.14	0.84±0.19	0.65±0.12	0.66±0.18	0.77±0.19	0.81±0.1	0.81±0.11	0.76±0.13
25	0.77±0.15	0.83±0.15	0.7±0.18	0.71±0.15	0.76±0.19	0.82±0.13	0.86±0.11	0.81±0.11
30	0.74±0.15	0.84±0.14	0.72±0.18	0.69±0.17	0.81±0.18	0.88±0.11	0.88±0.12	0.81±0.16
35	0.8±0.15	0.83±0.14	0.69±0.15	0.73±0.14	0.79±0.15	0.84±0.16	0.91±0.09	0.84±0.14
40	0.78±0.17	0.85±0.15	0.8±0.12	0.78±0.13	0.8±0.16	0.85±0.14	0.89±0.13	0.81±0.14
45	0.81±0.22	0.88±0.13	0.84±0.1	0.79±0.14	0.84±0.11	0.87±0.15	0.91±0.14	0.84±0.14
50	0.81±0.2	0.88±0.12	0.86±0.13	0.81±0.13	0.84±0.12	0.88±0.13	0.89±0.13	0.84±0.15
55	0.84±0.16	0.88±0.12	0.85±0.12	0.85±0.17	0.85±0.14	0.89±0.11	0.91±0.11	0.84±0.15
60	0.84±0.14	0.88±0.15	0.81±0.16	0.84±0.14	0.85±0.11	0.87±0.16	0.91±0.11	0.84±0.15
65	0.85±0.13	0.89±0.12	0.82±0.17	0.85±0.13	0.89±0.11	0.87±0.13	0.92±0.11	0.86±0.14
70	0.83±0.13	0.88±0.12	0.86±0.13	0.85±0.15	0.88±0.13	0.89±0.12	0.91±0.11	0.88±0.13
75	0.83±0.16	0.89±0.12	0.88±0.15	0.86±0.13	0.88±0.11	0.89±0.11	0.89±0.11	0.89±0.13
80	0.86±0.19	0.88±0.13	0.81±0.13	0.85±0.12	0.89±0.11	0.89±0.14	0.89±0.12	0.89±0.11
85	0.86±0.13	0.88±0.14	0.81±0.16	0.82±0.1	0.89±0.11	0.88±0.12	0.89±0.11	0.89±0.11
90	0.86±0.12	0.88±0.14	0.84±0.14	0.84±0.14	0.86±0.11	0.88±0.13	0.91±0.11	0.85±0.13
95	0.86±0.1	0.88±0.11	0.81±0.14	0.85±0.14	0.89±0.11	0.89±0.11	0.89±0.12	0.86±0.11
100	0.86±0.12	0.88±0.12	0.86±0.11	0.85±0.13	0.89±0.11	0.89±0.12	0.91±0.11	0.88±0.11
105	0.86±0.11	0.88±0.11	0.82±0.12	0.85±0.1	0.91±0.11	0.89±0.1	0.87±0.12	0.88±0.13
110	0.85±0.13	0.88±0.11	0.84±0.11	0.82±0.13	0.91±0.13	0.89±0.1	0.89±0.11	0.89±0.13
115	0.85±0.13	0.88±0.12	0.85±0.1	0.82±0.14	0.91±0.11	0.89±0.09	0.89±0.11	0.89±0.14
120	0.87±0.12	0.89±0.12	0.85±0.1	0.84±0.12	0.91±0.11	0.88±0.1	0.89±0.11	0.88±0.12
125	0.87±0.11	0.88±0.12	0.84±0.13	0.84±0.14	0.91±0.13	0.88±0.11	0.88±0.11	0.87±0.11
130	0.88±0.12	0.89±0.11	0.84±0.11	0.83±0.12	0.91±0.15	0.88±0.11	0.88±0.1	0.87±0.12
135	0.89±0.11	0.89±0.11	0.88±0.11	0.84±0.11	0.88±0.11	0.91±0.11	0.88±0.1	0.87±0.11
140	0.89±0.1	0.89±0.12	0.88±0.09	0.86±0.09	0.89±0.11	0.89±0.11	0.89±0.11	0.88±0.12
145	0.89±0.11	0.89±0.11	0.88±0.1	0.89±0.12	0.88±0.11	0.91±0.12	0.89±0.1	0.88±0.11
150	0.91±0.09	0.89±0.12	0.89±0.09	0.88±0.12	0.91±0.12	0.89±0.11	0.85±0.12	0.87±0.11

Table S3 - Detailed results of performance validation for ProstateGE dataset

# of features	t-score	Fisher score	Trace ratio	Multi SURF	ClearF normal	ClearF rbf	ClearF poly
5	0.79±0.18	0.92±0.2	0.88±0.08	0.86±0.2	0.86±0.12	0.92±0.2	0.88±0.2
10	0.81±0.19	0.87±0.11	0.92±0.11	0.84±0.2	0.93±0.09	0.94±0.19	0.94±0.2
15	0.87±0.25	0.92±0.2	0.91±0.21	0.87±0.2	0.93±0.08	0.91±0.09	0.92±0.09
20	0.86±0.21	0.86±0.21	0.83±0.14	0.86±0.08	0.94±0.09	0.93±0.08	0.93±0.08
25	0.92±0.2	0.92±0.13	0.83±0.11	0.85±0.2	0.92±0.19	0.92±0.09	0.93±0.09
30	0.9±0.11	0.91±0.11	0.86±0.12	0.86±0.22	0.87±0.2	0.91±0.09	0.92±0.08
35	0.91±0.1	0.91±0.1	0.91±0.1	0.92±0.2	0.91±0.19	0.91±0.11	0.93±0.09
40	0.91±0.11	0.92±0.11	0.9±0.11	0.86±0.1	0.92±0.1	0.91±0.19	0.92±0.09
45	0.92±0.13	0.91±0.1	0.86±0.1	0.87±0.11	0.87±0.19	0.86±0.19	0.92±0.2
50	0.91±0.11	0.92±0.11	0.9±0.1	0.92±0.19	0.93±0.09	0.89±0.09	0.92±0.1
55	0.92±0.11	0.93±0.11	0.92±0.11	0.92±0.08	0.92±0.09	0.92±0.19	0.91±0.2
60	0.9±0.1	0.92±0.11	0.9±0.11	0.87±0.2	0.86±0.09	0.9±0.1	0.91±0.11
65	0.92±0.1	0.92±0.1	0.91±0.1	0.86±0.11	0.91±0.1	0.92±0.14	0.91±0.2
70	0.86±0.11	0.91±0.11	0.91±0.1	0.86±0.09	0.92±0.1	0.92±0.19	0.9±0.11
75	0.91±0.11	0.9±0.1	0.93±0.08	0.86±0.2	0.92±0.19	0.91±0.1	0.89±0.2
80	0.91±0.11	0.91±0.1	0.91±0.11	0.86±0.19	0.92±0.09	0.9±0.2	0.89±0.19
85	0.92±0.11	0.93±0.11	0.93±0.08	0.92±0.08	0.92±0.09	0.92±0.11	0.85±0.09
90	0.92±0.09	0.87±0.08	0.92±0.11	0.85±0.11	0.93±0.09	0.94±0.19	0.92±0.09
95	0.92±0.11	0.93±0.11	0.92±0.08	0.86±0.19	0.93±0.19	0.92±0.09	0.9±0.19
100	0.93±0.09	0.93±0.09	0.92±0.1	0.87±0.19	0.93±0.09	0.92±0.11	0.91±0.1
105	0.93±0.08	0.91±0.11	0.86±0.08	0.88±0.09	0.93±0.09	0.91±0.11	0.91±0.1
110	0.9±0.09	0.91±0.08	0.92±0.08	0.86±0.09	0.93±0.08	0.92±0.09	0.92±0.1
115	0.91±0.08	0.94±0.11	0.93±0.08	0.85±0.1	0.93±0.09	0.92±0.08	0.91±0.11
120	0.9±0.09	0.92±0.09	0.92±0.1	0.9±0.2	0.93±0.11	0.92±0.1	0.92±0.11
125	0.9±0.11	0.92±0.09	0.92±0.1	0.92±0.09	0.93±0.08	0.9±0.08	0.92±0.1
130	0.91±0.08	0.92±0.09	0.94±0.11	0.85±0.08	0.92±0.08	0.92±0.08	0.9±0.09
135	0.93±0.09	0.91±0.11	0.92±0.09	0.94±0.08	0.93±0.08	0.93±0.08	0.91±0.09
140	0.93±0.11	0.9±0.09	0.91±0.11	0.87±0.08	0.9±0.2	0.92±0.07	0.9±0.1
145	0.91±0.09	0.92±0.11	0.93±0.08	0.87±0.08	0.93±0.19	0.93±0.09	0.9±0.1
150	0.92±0.08	0.91±0.09	0.93±0.08	0.93±0.2	0.93±0.08	0.92±0.08	0.92±0.1

Table S4 - Detailed results of performance validation for Leukemia dataset

# of features	mRMR	CMIM	t-score	Fisher score	Trace ratio	Multi SURF	ClearF normal	ClearF rbf	ClearF poly
5	0.93±0.09	0.92±0.1	0.95±0.15	0.93±0.1	0.9±0.15	0.9±0.14	0.96±0.14	0.89±0.13	0.93±0.1
10	0.96±0.15	0.92±0.06	0.93±0.09	0.96±0.07	0.96±0.1	0.96±0.07	0.96±0.07	0.92±0.09	0.97±0.06
15	0.94±0.14	0.96±0.1	0.93±0.1	0.92±0.09	0.94±0.1	0.93±0.07	0.97±0.06	0.94±0.1	0.93±0.1
20	0.93±0.07	0.93±0.1	0.96±0.07	0.94±0.1	0.96±0.06	0.93±0.1	0.94±0.1	0.96±0.07	0.96±0.1
25	0.94±0.07	0.97±0.07	0.94±0.07	0.94±0.1	0.96±0.06	0.94±0.1	0.93±0.1	0.94±0.1	0.96±0.1
30	0.96±0.06	0.97±0.1	0.96±0.07	0.94±0.1	0.94±0.1	0.94±0.1	0.97±0.06	0.96±0.07	0.94±0.1
35	0.96±0.14	0.96±0.07	0.94±0.06	0.94±0.1	0.94±0.1	0.94±0.1	0.94±0.1	0.96±0.07	0.94±0.1
40	0.96±0.14	0.97±0.1	0.93±0.06	0.94±0.1	0.94±0.1	0.94±0.1	0.94±0.1	0.96±0.1	0.94±0.1
45	0.96±0.14	0.97±0.07	0.93±0.06	0.94±0.09	0.94±0.1	0.96±0.07	0.97±0.07	0.94±0.07	0.96±0.06
50	0.96±0.14	0.97±0.07	0.92±0.06	0.94±0.07	0.94±0.06	0.94±0.1	0.96±0.06	0.96±0.1	0.97±0.07
55	0.96±0.14	0.97±0.07	0.93±0.06	0.96±0.07	0.94±0.1	0.94±0.1	0.96±0.1	0.97±0.06	0.97±0.06
60	0.96±0.14	0.97±0.07	0.97±0.06	0.97±0.07	0.96±0.07	0.94±0.1	0.96±0.06	0.96±0.07	0.94±0.1
65	0.97±0.1	0.97±0.07	0.96±0.06	0.96±0.07	0.96±0.07	0.94±0.1	0.97±0.06	0.94±0.07	0.94±0.1
70	0.97±0.1	0.97±0.06	0.94±0.06	0.96±0.06	0.96±0.06	0.96±0.07	0.96±0.07	0.96±0.06	0.97±0.1
75	0.97±0.06	0.96±0.06	0.97±0.07	0.96±0.07	0.97±0.07	0.96±0.07	0.94±0.1	0.97±0.07	0.93±0.06
80	0.96±0.1	0.97±0.07	0.94±0.06	0.96±0.07	0.96±0.07	0.96±0.07	0.97±0.1	0.96±0.07	0.97±0.07
85	0.97±0.1	0.97±0.07	0.96±0.06	0.96±0.07	0.96±0.07	0.96±0.07	0.96±0.07	0.97±0.06	0.97±0.1
90	0.96±0.1	0.96±0.07	0.96±0.06	0.96±0.07	0.96±0.07	0.96±0.07	0.95±0.1	0.97±0.06	0.96±0.06
95	0.97±0.1	0.97±0.07	0.96±0.07	0.96±0.07	0.97±0.06	0.96±0.07	0.97±0.06	0.97±0.06	0.96±0.07
100	0.96±0.1	0.97±0.07	0.97±0.06	0.96±0.07	0.99±0.05	0.96±0.07	0.93±0.07	0.97±0.06	0.96±0.07
105	0.97±0.06	0.97±0.07	0.97±0.06	0.99±0.05	0.97±0.06	0.96±0.07	0.94±0.07	0.97±0.06	0.96±0.07
110	0.97±0.06	0.96±0.07	0.97±0.06	0.97±0.06	0.97±0.06	0.96±0.07	0.94±0.07	0.97±0.06	0.95±0.07
115	0.97±0.06	0.96±0.06	0.97±0.06	0.97±0.06	0.97±0.06	0.96±0.07	0.96±0.07	0.97±0.06	0.95±0.07
120	0.99±0.06	0.97±0.06	0.97±0.06	0.97±0.06	0.96±0.07	0.96±0.07	0.94±0.07	0.97±0.06	0.95±0.07
125	0.97±0.06	0.99±0.06	0.97±0.06	0.97±0.06	0.97±0.06	0.96±0.07	0.96±0.07	0.97±0.05	0.96±0.06
130	0.96±0.06	0.96±0.06	0.97±0.06	0.97±0.06	0.97±0.06	0.96±0.07	0.96±0.07	0.99±0.05	0.94±0.07
135	0.97±0.05	0.96±0.06	0.99±0.06	0.96±0.07	0.99±0.05	0.96±0.07	0.94±0.07	0.99±0.05	0.97±0.06
140	0.97±0.05	0.95±0.07	0.99±0.06	0.99±0.05	0.99±0.05	0.96±0.07	0.96±0.07	0.99±0.05	0.96±0.06
145	0.99±0.05	0.96±0.07	0.99±0.07	0.99±0.05	0.97±0.06	0.97±0.07	0.94±0.07	0.97±0.05	0.97±0.1
150	0.99±0.05	0.97±0.07	0.99±0.07	0.99±0.05	0.99±0.05	0.96±0.07	0.96±0.1	0.99±0.05	0.96±0.06

Table S5 - Detailed results of performance validation for TOX171 dataset

# of features	Fisher score	Trace ratio	Multi SURF	ClearF normal	ClearF rbf	ClearF poly
5	0.6±0.13	0.5±0.13	0.5±0.08	0.5±0.11	0.5±0.18	0.52±0.14
10	0.67±0.14	0.6±0.1	0.64±0.09	0.68±0.12	0.59±0.09	0.5±0.13
15	0.65±0.17	0.63±0.15	0.73±0.12	0.73±0.12	0.57±0.1	0.56±0.1
20	0.64±0.15	0.63±0.13	0.81±0.09	0.77±0.12	0.63±0.12	0.63±0.12
25	0.65±0.14	0.71±0.14	0.82±0.09	0.81±0.1	0.67±0.1	0.69±0.1
30	0.67±0.11	0.71±0.11	0.82±0.07	0.81±0.1	0.67±0.08	0.68±0.13
35	0.7±0.11	0.73±0.11	0.86±0.1	0.83±0.09	0.68±0.13	0.74±0.13
40	0.67±0.1	0.74±0.12	0.85±0.08	0.85±0.12	0.7±0.11	0.72±0.09
45	0.71±0.09	0.74±0.11	0.86±0.1	0.87±0.1	0.75±0.12	0.75±0.13
50	0.74±0.09	0.77±0.09	0.88±0.1	0.89±0.12	0.76±0.13	0.75±0.13
55	0.74±0.09	0.78±0.09	0.89±0.08	0.9±0.11	0.75±0.14	0.77±0.12
60	0.78±0.1	0.78±0.07	0.92±0.08	0.9±0.09	0.82±0.15	0.78±0.11
65	0.81±0.08	0.81±0.1	0.89±0.08	0.9±0.1	0.81±0.09	0.78±0.11
70	0.8±0.13	0.8±0.12	0.91±0.08	0.91±0.1	0.82±0.07	0.76±0.11
75	0.79±0.11	0.8±0.12	0.92±0.09	0.92±0.1	0.82±0.09	0.76±0.11
80	0.79±0.1	0.81±0.1	0.92±0.08	0.94±0.08	0.83±0.09	0.75±0.11
85	0.84±0.1	0.82±0.1	0.92±0.07	0.94±0.09	0.86±0.11	0.79±0.06
90	0.82±0.11	0.83±0.11	0.94±0.07	0.93±0.1	0.87±0.09	0.82±0.08
95	0.83±0.11	0.82±0.11	0.93±0.08	0.92±0.1	0.88±0.09	0.79±0.09
100	0.83±0.11	0.83±0.12	0.95±0.07	0.93±0.1	0.87±0.08	0.82±0.07
105	0.81±0.11	0.82±0.12	0.95±0.08	0.93±0.09	0.89±0.06	0.81±0.08
110	0.81±0.14	0.79±0.1	0.95±0.08	0.95±0.09	0.89±0.06	0.82±0.08
115	0.84±0.11	0.81±0.12	0.95±0.07	0.94±0.09	0.89±0.07	0.82±0.07
120	0.81±0.13	0.79±0.12	0.95±0.07	0.95±0.08	0.91±0.08	0.84±0.06
125	0.82±0.11	0.79±0.1	0.95±0.07	0.95±0.08	0.89±0.08	0.85±0.07
130	0.84±0.12	0.81±0.1	0.94±0.07	0.96±0.07	0.9±0.08	0.87±0.08
135	0.83±0.1	0.81±0.09	0.95±0.07	0.96±0.07	0.91±0.06	0.87±0.08
140	0.83±0.09	0.84±0.08	0.95±0.07	0.96±0.07	0.92±0.08	0.87±0.09
145	0.85±0.09	0.85±0.09	0.94±0.08	0.96±0.07	0.94±0.08	0.88±0.08
150	0.88±0.1	0.88±0.1	0.97±0.07	0.96±0.07	0.93±0.08	0.85±0.09

Table S6 - Detailed results of performance validation for TCGA dataset

# of features	Fisher score	Trace ratio	Multi SURF	ClearF normal	ClearF rbf	ClearF poly
5	0.78±0.09	0.8±0.09	0.83±0.08	0.83±0.08	0.8±0.08	0.79±0.07
10	0.82±0.09	0.84±0.07	0.83±0.09	0.83±0.08	0.85±0.07	0.85±0.07
15	0.84±0.08	0.83±0.09	0.82±0.09	0.86±0.07	0.86±0.07	0.86±0.08
20	0.83±0.08	0.85±0.08	0.83±0.09	0.84±0.07	0.86±0.06	0.87±0.06
25	0.85±0.08	0.85±0.07	0.83±0.09	0.85±0.07	0.86±0.06	0.86±0.06
30	0.85±0.07	0.85±0.08	0.84±0.1	0.84±0.07	0.86±0.06	0.86±0.07
35	0.84±0.09	0.85±0.08	0.84±0.09	0.84±0.07	0.85±0.07	0.86±0.08
40	0.84±0.08	0.84±0.09	0.83±0.08	0.86±0.08	0.85±0.06	0.86±0.08
45	0.85±0.09	0.85±0.1	0.83±0.1	0.85±0.08	0.86±0.07	0.86±0.06
50	0.86±0.08	0.86±0.09	0.84±0.09	0.86±0.08	0.86±0.07	0.85±0.07
55	0.85±0.07	0.85±0.08	0.84±0.09	0.85±0.07	0.86±0.08	0.85±0.08
60	0.86±0.07	0.85±0.08	0.83±0.09	0.86±0.07	0.85±0.07	0.85±0.08
65	0.85±0.09	0.85±0.07	0.82±0.09	0.85±0.07	0.86±0.08	0.86±0.08
70	0.84±0.08	0.85±0.09	0.82±0.09	0.86±0.09	0.86±0.07	0.85±0.07
75	0.83±0.1	0.85±0.07	0.83±0.09	0.85±0.09	0.85±0.08	0.85±0.08
80	0.86±0.08	0.85±0.09	0.83±0.1	0.84±0.07	0.86±0.08	0.84±0.09
85	0.84±0.08	0.84±0.09	0.82±0.1	0.85±0.07	0.86±0.08	0.85±0.07
90	0.84±0.09	0.85±0.09	0.82±0.09	0.85±0.07	0.84±0.08	0.85±0.07
95	0.83±0.09	0.86±0.09	0.81±0.1	0.86±0.07	0.85±0.08	0.85±0.08
100	0.84±0.09	0.85±0.08	0.82±0.09	0.86±0.07	0.85±0.08	0.85±0.07
105	0.84±0.09	0.85±0.08	0.82±0.09	0.85±0.08	0.85±0.07	0.85±0.08
110	0.84±0.09	0.85±0.09	0.83±0.09	0.85±0.07	0.85±0.08	0.84±0.07
115	0.84±0.09	0.84±0.08	0.83±0.09	0.85±0.07	0.84±0.08	0.85±0.09
120	0.85±0.08	0.85±0.08	0.83±0.09	0.85±0.07	0.84±0.07	0.83±0.09
125	0.84±0.08	0.85±0.08	0.83±0.08	0.84±0.07	0.83±0.07	0.84±0.07
130	0.84±0.09	0.85±0.08	0.83±0.09	0.85±0.07	0.83±0.06	0.84±0.07
135	0.84±0.09	0.85±0.08	0.82±0.09	0.85±0.08	0.84±0.07	0.84±0.08
140	0.84±0.08	0.85±0.09	0.82±0.09	0.85±0.08	0.85±0.08	0.83±0.08
145	0.83±0.08	0.85±0.09	0.82±0.08	0.85±0.08	0.83±0.08	0.83±0.08
150	0.83±0.08	0.84±0.09	0.82±0.08	0.85±0.09	0.84±0.08	0.83±0.08
